# Cause and Treatment of the Uric Acid Diathesis

**Published:** 1896-03

**Authors:** N. A. Olive

**Affiliations:** Waco, Texas


### TEXAS MEDICAL JOURNAL.

ESTABLISHED JULY, 1885.

PUBLISHED MONTHLY.—SUBSCRIPTION $2.00 A YEAR.

VOL. XI. AUSTIN, MARCH, 1896. NO. 9.

### Original Contributions.

For the Texas Medical Journal.

### CAUSE AND TREATMENT OF THE URIC ACID DIATHESIS.

BY N. A. OLIVE, M. D., WACO, TEXAS.

[Read before the Central Texas Medical Society, Jan. 14, 1896.]

THE importance of this subject has been dwelt upon by pathologists for many years, and uric acid as a factor in the causation of a limited number of diseases has been fully recognized. But the knowledge that it is a factor in the ætiology of many diseases of hitherto unknown pathology, has of recent years proven a revelation to the medical world.

To Alexander Haig, of London, the profession is indebted for much of original investigation along this line. And his work, though perhaps incorrect in some of his theories, in the main is correct, and is well worth the perusal of any busy practitioner.

Uric acid was discovered by Scheele in 1776, and was at first thought to be solely a constituent of urinary calculi; hence the term lithic acid applied to it. In 1797 Woolaston showed that gouty concretions were composed of sodium urate.

In 1848 Garrod claimed that in true gout an excess of uric acid exists in the blood prior to and at the period of the attack. It is claimed by chemists that by oxidation a molecule of uric acid can be split up in a molecule of non-nitrogenous acid and two molecules of urea, and it has been assumed that when the process of oxidation is imperfectly performed within the body, free uric acid will be found in excess in the urine. It has been demonstrated, however, that uric acid is not a necessary antecedent of urea, which is largely formed from kreatin in muscle and lucin and other bodies in the alimentary canals. Uric acid in a healthy body is formed in much less quantity than was formerly supposed, and in disease the large amount excreted is indicative of cumulative quantity, the result of previous faulty excretion, rather than greatly increased formation. Its deposit and retention in tissues being the result of its extreme insolubility. It is soluble in water in proportion of 1 to 15,000. Haig speaks of the uric acid daily excreted from the urine coming from three sources:

"1. The uric acid or urate formed out of nitrogenous substances along with a certain amount of urea, probably in the proportion of one of uric acid to 35 of urea. 2. Uric acid, urates, or other members of the xanthin group, as caffeine and other vegetable alkaloids introduced with the food. 3. Uric acid or urates previously stored in the body, which pass into the blood whenever the alkalinity of that favors their solution in it."

Haig, however, uses the term uric acid in an inclusive manner, comprehending uric acid and its salts, and he states that "when in 24 hours urine, uric acid is present in the relation to urea of 1 to 35, we conclude that this uric acid was formed along with the urea of that day and passed directly down the ureter into the urine and that there was no uric acid furnished direct from the food, or from the stores in the body. If any uric acid was introduced in the food, this was not allowed to remain in the blood, because of the conditions of that fluid to its solubility; and for the same reason no uric acid was taken up from the stores in the body. If, however, uric acid was excreted in the relation of 1 to 20 or 25 of urea we conclude that the excess over formation (1 to 35), is made up from uric acid introduced with the food, or taken up from the deposits in the body. Under these conditions it is quite easy to understand why all substances which form compounds with uric acid, or increase its solubility in the blood, should increase its excretion; and why all substances which form insoluble compounds with it, or diminish its solubility in the blood, should diminish its excretion. If, on the other hand, the excretion of uric acid is very low, having the relation to urea of 1 to 45 or 1 to 50, we must conclude that some of the uric acid, formed on this day, met with conditions in the kidney which were unfavorable to its solubility, and was retained there instead of passing at once down the ureter, and we know that the kindeys may contain an amount which varies from a small percentage up to a quantity which is plainly visible to the eye.''

Haig's conclusions, therefore, after extensive experimentation, are: that the formation of uric acid in the body is practically constant, while excretion varies partly with the conditions affecting its solubility in the blood, and partly with the quantity introduced in the food, or previously retained in the body, that is, the quantity available for solution. He states that it is "obviously then in our power to control the excretion of uric acid by controlling the conditions affecting its solubility, and also the quantities directly introduced iu the food.''

Perhaps some method of determination of uric acid and urea, especially as to quantity, will be of interest at this particular time. Purdy speaks of Heintz's method as sufficiently accurate for most clinical purposes. "Take 200 cubic centimetres of urine, and add to it 10 cubic centimetres of hydrochloric acid. Let it stand for twenty-four hours, iu a cool room. Collect the precipitated uric acid crystal on a previously weighed filter, and wash with cold distilled water. Dry the filter and uric acid crystals in a dessicator, and weigh. By subtracting the weight of the filter, the result will be the weight of uric acid in 200 cubic centimetres of urine. If albumen is present, it should first be coagulated by a few drops of acetic acid and heat, and then filtered off.'' And as to the estimation of urea, Leibig's method with mercuric oxide, is of good general utility. But the clinician can select his own method of determining the quantity and relation of the two constituents of the urine. Haig says, ''From the clinical history of the uric acid headache, we learn that at the time of the attack, when there is excessive uric acid in the blood and in the urine, the pulse is generally slow, and of high tension. It appears that this is due to contraction of the arterioles and capillaries, of which there is abundant evidence in the cold skin and extremities that accompany the headache. And a very little experimentation will, I believe, suffice to convince any one that contraction of the arterioles varies directly with the uric acid that is circulating in the blood. And this contraction of arterioles will produce two results: (1) A rise of blood pressure in the heart and great vessels on the proximal side of the obstructed vessels—high arterial tension, and (2) a deficient circulation and interchange between the blood and the tissues in and on the distal side of the obstructed vessels. It follows from this that all drugs, and disease processes, which diminish the excretion of uric acid and clear it out of the blood, will lower arterial tension, and improve the circulation through all the organs and tissues of the body. Drugs have already been mentioned; and the most important disease process which has this effect, is fever, which diminishes the alkalinity of the blood, and thus prevents its holding much urate in solution. Conversely, drugs and disease processes which increase the excretion of uric acid and bring it through the blood in increased quantity, will raise arterial tension, and diminish or hinder the circulation through all the organs and tissues of the body. The most important disease processes which produce this effect are Bright's disease, post-febrile conditions, dyspepsia, spleenic leucocythæmia, marasmus, and the cachexia of new growths, etc., for in all these conditions of low or failing nutrition there is commonly some increase of the alkalinity of the blood, rendering it a more than usually good solvent of uric acid; and in post-febrile conditions not only does the blood thus become a good solvent of uric acid, but a large supply of uric acid is present, ready to be dissolved, namely, that which was held back and retained during the low alkalinity of the acute febrile state. Now, other things being equal, a dilation of the arterioles, of the kidneys, means a free flow of urinary water, and their contraction means scanty water. For this reason, the urine is scanty (30 cc. per hour) during the uric acid headache (migraine), and profuse (60–160 cc. per hour) during the minus excretion of urates, which precedes and follows it. And not only is this true in pathology, but so constant is the relation of contracted arterioles to uric acid, that is physiology also, in every one, from hour to hour and day to day, the urinary water varies inversely as the uric acid excreted along with it; or, to state it as accurately as possible, other things being equal, the more the excretion of uric acid exceeds the relation to urea of 1 to 35, the more scanty will be the correspondingly hourly or daily excretion of water, and *vice versa;* because, as we have seen from previous arguments, the uric acid which is excreted in excess of formation (1-35) probably passes through the blood on its way to the kidneys."

Haig claims that all diseases, except rheumatism and gout, attributable to uric acid, are due to its effects on arterial tension and on the intersticial circulation of organs and tissues throughout the body. Thus, headache, fits, mental depression, and bad temper, as also vertigo, and a host of minor functional disturbances, represent the effects of high arterial tension on the intracranial circulation. "In saying this, as in all I shall have to say, I wish it to be understood that while I consider uric acid to be a cause, often the most important cause, of these disturbances, I should never go so far as to put it down as the only cause; and in the chapters which treat of the cerebral circulation, it will, I hope, be quite evident that other causes may produce similar alterations in the cerebral circulation, and therefore similar symptoms. The well known symptoms of high arterial tension represent the effects of uric acid in contracting the arterioles and raising the pressure in the arterial system behind them. Asthma and chronic bronchitis, again, probably represent some of the effects of high arterial tension and contracted arterioles on the bronchial and pulmonary circulation, and this probably explains the relations so often seen between these diseases on the one hand, and gout and chronic Bright's disease on the other. Dyspepsia may undoubtedly be produced by contracted arterioles in the stomach and intestines, which inhibit gastro-intestinal digestion and allow putrefactive processes to take their place, and this again will explain at least part of the relationship between dyspepsia and such troubles as headache, epilepsy, and mental depression; or between dyspepsia and Raynaud's disease, or paroxysmal hæmoglobinuria. In the liver again, just as in the stomach or intestines, we may obviously get more or less stasis or congestion, as a direct or indirect result of the circulatory changes produced by uric acid. Precisely the same thing applies, word for word, to the kidneys, and here we may have a very obvious and tangible result of the changes so produced in its circulation, in the fluctuations in the urinary water previously referred to. The same thing is seen in the salivary, and probably in all other glands; and, in accordance with this, when the uric acid headache is present, and the urinary water is scanty, the mouth also tends to be dry or sticky, and the saliva is scanty; and when the headache has been cured by acids, opium, mercury, iodides, or any of the drugs which clear the uric acid out of the blood, not only is there a diuresis, but the saliva also becomes relatively profuse." Now, without doubt the effect of uric acid upon the circulation of the blood is of vast importance, and Haig has well said: "If my premises are good, and my deductions sound, and if uric acid really influences the circulation to the extent which I have been led to believe that it does, it follows that uric acid really dominates the functions, nutrition and structure of the human body to an extent which has never yet been dreamed of in our philosophy, and in place of affecting the structure of a few comparatively insignificant fibrous tissues, in which it is found after death, it may really direct the development, life history, and final decay and dissolution of every tissue, from the most important nerve centers and the most active glands to the matrix and the nails and the structure of the skin and hair."

We have quoted rather freely from Haig in order to arrive at a more perfect understanding of the means by which uric acid produces certain pathological conditions, and thereby be able to better determine the causes and treatment of the uric acid diathesis.

Now among the diseases most likely to favor an accumulation of uric acid in the body are, the acute febrile disturbances, raising the acidity of the blood, rendering the uric acid insoluble, and favoring its deposit in the tissues. Kidney disease may as well favor the accumulation of uric acid in the body, as to be the direct result of the diathesis. Any skin disease whereby the function of this organ is disturbed or arrested, may favor the development of the conditions, or it may also be dependent upon the same. As also is acid dyspepsia as often the cause, as a result of uric acid in the system. Inactivity of the liver and improperly performed functions of the intestines, with constipation and other evil consequences are among the most important ætiological factors. Pulmonary or cardiac diseases, with deficiency of oxygen, with general inactivity of all the organs, are important causes, whether the uric acid is a sub-oxidation product or otherwise. Climate has much to do with a development of this diathesis; especially is this true of the cold or wet weather which arrests the function of the skin and respiratory mucous membrane to a certain extent. Season, as it contributes to humidity of the atmosphere, whether hot or cold, is a factor of no mean import. Diet, as meat and meat broths, extracts, etc., as formerly suggested, and starchy foods and sweets, as also acids, malt liquors, ale, porter or beer, and wines, contribute largely to this trouble. Haig claims that among the poor in non-meat-eating districts, rheumatism and gout never occur. Inactivity, want of general muscular exercise, is perhaps of first importance ætiologically. Occupation, in so far as it contributes to any of the above causes, is influential in the production of the uric acid diathesis. The habitual use of tobacco is potent in causing the accumulation of uric acid in the system.

TREATMENT:—In treatment certain prophylaxis is of the greatest importance. Alcoholic drinks are to be sedulously avoided. Meats are to be taken seldom and in small quantities; tobacco stopped. The immunity to the uric acid trouble enjoyed by sea-faring men is said to be due to the full amount of salt which they consume, and which retards the precipitation of free uric acid in the body. The blood brings the Na. Cl. into the peptic glands, and the H. 2 o. of the stomach passes through the wall of the gland by osmosis, and the following chemical change takes place. The Na. Cl.+H. 1 O=Na. Ho.+H. C. 1. The H. C. 1 gives the acidity to the gastric juice and the Na. Ho. (sodic hydrate) unites with the C. O. 2 of the blood to form the carbonate of sodium, which together with the sodium phosphate maintains the alkaline condition of the blood. We will then advise the free use of sodium chloride with the food. A milk and vegetable diet with some fruit and an occasional egg, and a fish dinner now and then, including with the vegetable diet the various breads. This diet may generally be adhered to with reasonable safety. All things that may or may not be taken can not be mentioned in so brief a paper. A remedy that is both prophylactic and curative is good, pure, hot water, applied externally. The hot waters of Waco can not be excelled for this purpose. Exercise, not too violent, must be enjoined, and this must be kept up regularly in a systematic manner. Acute febrile troubles of uric acid origin may be successfully met by salicylic acic, salol, salicylate of quinia, or cichonidia, or a combination of salicylitic acid with a good diuretic. The following has been useful in my practice:

℞. (3) Acid salicylic.........ʒiij

(5) Potass acet............ʒv

(5) Aq. dist...........qs. ℥jv

M. ft. Sol. Sig.

Take a teaspoonful in water every four hours.

Piperazine has received its share of the praise of late as a remedy for uric acid. But it is claimed that if given when the kidneys are diseased it will produce or increase albuminuria. I have no practical experience with the drug, and know little of its advantages or disadvantages. Salicylate of sodium should have been mentioned earlier; but this or any of the sodium preparations should not be persisted in too long, as the chalky concretions of gout are composed of sodium urate and this remdy and uric acid might form this combination. The salts of potassium are of great value, and magnesia is also of utility. A combination that I consider as efficient as any that can be given in a subacute or chronic condition of uric-acid-æmia [?] is

℞. (2) Potass. bicarb......... ʒij

(½) Magnesia carb.........ʒss

(½) Pulv. Rhei...... . . ʒss

(1) Tr. colchii. sem........ʒj

(2) Spts. amm. arom......ʒij

(8) Aq. menth. pip..q. s. ℥viij.

M. ft. sol. Sig.

Take one to two table spoonfuls in water three times daily.

The coal tar preparations and opiates are to be avoided as they produce vaso-motor paralysis, with consequent dilation, and instead of favoring excretion retard it. Hence if addicted to an opiate its use must be discontinued before anything can be expected of treatment. Purgatives and laxatives must be employed as occasion seems to demand. Iodide of potassium is sometimes of use when almost all other remedies have been used with little apparent benefit. Ammonia preparations are said to be sometimes useful. But if I were consulted for this condition, the things I would insist upon as of first importance, would be great care as to diet, strict attention to habits of cleanliness, frequent baths and large draughts of hot water and uniformity and warmth of dress, and habits of exceeding regularity, not forgetting the necessary amount of exercise.

### DISCUSSION ON DR. OLIVE'S PAPER (CAUSES AND TREATMENT OF THE URIC ACID DIATHESIS).

DR. YOUNG:—I do not know what can be said of that paper. Dr. Olive has told us all about uric acid from 1776 to the present date. He has stated the causes as well as the treatment. Of course, we have used the treatment for several years, that is to say—the alkali treatment—as a uric acid solvent for rheumatism and gout, but until recently we have not used it for asthma. It was called to our attention by our friend, Dr. Hunter, and since that time I have had very happy results in several cases with alkalithia treatment, and I have had good results in every case.

DR. BLAILOCK:—I had thought that I would spend a part of one day in a medical association without saying anything, but it seems that some of you have determined otherwise. I was very much interested in Dr. Olive's very exhaustive paper, and I listened to it from beginning to end with an increasing interest and was sorry that he quit, because I think it is altogether a good paper.

Now there are some points that cause me to shake a little. He gave us a history of it which is contemporaneous with the starting of this republic, about 1776. He also tells us that the use of tobacco is conducive to the uric acid diathesis. Now, I must confess that I am skeptical of a substance as universally employed as tobacco producing as grave a disease as the uric acid diathesis. As Dr. Hunter will tell you, we can not breathe, we have the asthma from the uric acid diathesis; we have gout from the uric acid diathesis; we have bilious attacks from the uric acid diathesis; and we have nearly all the ills that flesh is heir to from that uric acid diathesis. Now, if that can be produced from tobacco, this must be a much afflicted community, for you can not see a boy after he leaves his mother's lap who has not a cigarette in his mouth, and when he grows up to be a larger boy he has a cigar in his mouth. Now, it does seem to me that the uric acid diathesis is not caused by tobacco. Do not understand me that I am trying to take up for tobacco, because I have not a particle of sympathy for it. I think it is a useless and nasty habit; but we see men who have lived to be octogenarians, and I have in mind an old gentleman who died not long ago at the advanced age of ninety-nine, who had used tobacco all his life, and he never had but one or two symptoms in his life that required a doctor; he never had the uric acid diathesis in his life, and you call to mind numbers of others.

I will just say that I appreciate the paper, and in the main it is strictly correct, and it meets with my approbation; but I do not know so much about tobacco.

Dr. Howard:—I am an ardent believer in the uric acid diathesis. I have seen many instances of illness where I thought that the presence of uric acid in the system could explain the causes. I agree with Dr. Blailock that tobacco is not responsible for nearly all the ills. I think it is contributory in its influence to the accumulation of uric acid in the system, by causing an acidity of the blood and causing a deposition of uric acid in the joints, liver, spleen and other organs. It is injurious that far. I think that any acid taken into the blood, except it be perhaps the lighter vegetable acids, is deleterious in that respect, if kept up for any length of time. Acid taken into the blood rapidly clears the blood of uric acid, but it causes a deposition of uric acid in joints and liver and other ogans of which I have spoken. Contrary to Dr. Olive's paper I will state that my opinion will run counter to it so far—that quinine and almost all of the alkalies cause this acidity in the blood and cause a deposition of uric acid in the joints. Haig very clearly sets that forth, that quinine, morphine, and all the allied alkalies cause that; and caffeine he speaks of very markedly as causing it when used in excess. And I have seen several instances of the clearing of the blood of uric acid through or by giving salicylate of soda; a rapid diminution of all the symptoms, and the disease itself rapidly disappear. I think that gout itself can be cured by salicylate of soda, with those alkalies, if kept up for a sufficient length of time, if afterwards proper care is used in diet and exercise. But whenever we consume an excessive amount of animal food —meats,—and take plenty of wines and stimulants, and neglect to take proper exercise, we may expect to have rheumatism or gout, or some allied disease. About four years ago, I think it was, I reed a paper here on "Rheumatism." I made a statement then that I have never seen corroborated until about six months ago I saw it in the JOURNAL, and that was that salt was almost a preventive, if not a preventive against rheumatism, and had never seen a man who had used salt in his food to excess or in large amounts who had ever suffered with rheumatism. My attention was called to the fact one day by a gentleman who was buying some butter. His wife was a great sufferer from rheumatism. He tasted several specimens of butter, and said "My wife will not eat anything of that kind—that has salt in it." The idea struck me then, that perhaps that was the cause of this lady's suffering from rheumatism. I commenced to investigate the subject, and my experience since then has been that people who use salt in large quantities do not suffer with any form of rheumatism. Therefore I think that salt with our food is one of the greatest prophylactics against rheumatism, tonsilitis, bronchitis, in fact, all diseases due to this diathesis, of anything we can possibly use.

DR. WEATHERED:—I do not think I remember having heard a paper as lengthy as the doctor's was that kept the attention of all present so well. It seems that all present were equally interested with myself. I would like to ask a question in regard to the treatment. As to the effect of uric acid in causing contraction of the arterioles, I agree with the doctor,—but in speaking of the treatment, he says that the coal tar preparations should not be used, because they *dilate* the arterioles. It occurs to me that perhaps, in the main, that may be correct, but are there not cases where those coal tar preparations would be beneficial, for the reason that they *do* dilate those arterioles, and especially in those cases of asthma that are now supposed to be caused by the urid acid diathesis?

DR. GRAVES:—I listened with interest to the doctor's paper, and also to the discussions which we have heard, because this subject of uric acid diathesis has been one of more consideration to me lately than almost anything that has engaged my attention at all. There is no doubt in my mind but what a new and important field is developing, and very important researches are being made in this diathesis, stimulated by Haig's work and experiments, and his book, because they are being more widely read by physicians at large; and if one of you ever suffers with a uric acid storm in his system, or a severe bilious attack, and will read Haig's articles on that, you will get a photographic account of yourself that is just as good as any photographer in this or any other city can produce. Unfortunately, I am a sufferer from the uric acid diathesis, if bilious attacks, so-called, are one of the manifestations of that disease, and I had never been satisfied upon that point until I read Dr. Haig's book. I confess to you that he described my symptoms more accurately than I can do myself, and I have understood from that time, and have tried some of the experiments that he tried upon himself. He was a great sufferer from migraine. I can, within seventy-two hours, produce a bilious attack in myself, or, what I more properly term, according to him, a uric acid storm, by confining my diet almost exclusively to butcher's meats, beefsteak and roast and pork and coffee and tea, and leaving off the vegetable and non-nitrogenous foods, drinking very little water and taking very little exercise. If you will remember, in all these discussions it is not so much the manufacture of uric acid in the system as it is the excretion of urid acid in its proportion to the normal urea that is excreted through the system and through the kidneys. The researches of Dr. Haig, and many others, have demonstrated that the natural and normal proportion is about one to thirty-three or thirty-five; they vary within those limits; but if that normal ratio of excretion is kept up in the system by means of the proper regulation of the diet, or proper exercise, or by the use of any remedies that hold the uric acid in solution, or promote its excretion, then you do not have any manifestation of the uric acid diathesis. There are some things that occur, in our own experiences and observations, that ought to cause us to think along this line. This is a country that, as a rule, is free from gout. I venture to say that, among this large number of practitioners here, very few of you have a case of gout on hand right now, or have treated one for the past year. That is merely an illustration of the rarity of that disease here. Some of you may have seen a case, but certainly gout is a rare disease here. Now, why, if gout is produced by the uric acid diathesis? There are certainly a large number of men in this community who are sufferers from the uric acid diathesis. If the so-called "high living" is the causative factor alone of gout, then there are a large number of people, like my friend, Dr. Hengst, over there, that ought to have gout. There is something beyond all that in the uric acid diathesis, that neither Mr. Haig, nor any one else, has ever suggested, but which perhaps their investigations will yet lead to.

But referring again to the uric acid diathesis in its manifestation of a uric acid storm or a bilious attack, you will start with a coated tongue, a slightly bad taste in the mouth, especially a brackish taste in the morning. There will be a yellowish white coat on the tongue, perhaps immediately on the rear. Your appetite will begin to fail a little, your head will feel a little cloudy and a little heavy and congested, and you will find yourself getting cold in the extremities; your feet and your hands feel cold, and you have a languid feeling that it is impossible to describe, but once felt can always be recognized again. If you will note, during the prevalence of this severe headache there is usually a little nausea, or a good deal of nausea, present at that very time. The excretion from the kidneys is scanty. It is high colored; it is concentrated; the specific gravity is increased, but the quantity of water is decreased. The pulse is increased in its tension and volume, and decreased in its frequency. You may take a remedy, and you may take an alkali, and an alkali won't relieve that very promptly. An acid will relieve that then more readily and more promptly than alkali, but if you will vomit the patient freely, and remove from his system every exciting or irritating substance there, wash out his bowels freely, stimulate the circulation, and give an alkali, your patient will be relieved rather promptly. An opiate will do it, but in my own personal experience, five to ten grains of antikamnia will relieve me of one of those uric acid headaches more readily than any other remedy I have ever taken. Now, as I say, I can produce a uric acid storm in my system in seventy-two hours, and I can go for three weeks, or six weeks, or two months, without a single symptom manifesting itself, by the proper regulation of my diet. And a very important factor in connection with the diet is the regulation of the exercise. It is usually those people who eat heartily, whose bowels are constipated, whose skin perhaps is inactive, whose eliminative organs are not acting properly, and who do not take much exercise, who eat a large quantity of the nitrogenous foods, that have rheumatism, or have the other uric acid manifestations.

Now, in prescribing a proper diet and also a proper habit and regimen for the patient, you must not cut the patient off from the meat products entirely, because you will give him fish and fowl, and fish and fowl both contain more albumen than any butcher's meat in town; but the reason you give him fish and fowl is because he won't live on them all the time, and he will eat meat all the time. He will eat a steak for breakfast, a roast for dinner and a steak for supper, perhaps, and he will drink coffee for breakfast and dinner, and perhaps tea for supper; but you put him on a fish diet and keep him on that, and he will have uric acid manifestations, too. You put him on a fowl diet or a bird diet, which is worse (they contain more nitrogen than the ordinary meat products of the butcher shops), he will have gout and rheumatism, too. I believe that the proper amount of exercise, the proper elimination through the skin and through the kidneys and bowels, have the most important influence in regard to the development of these uric acid manifestations; and if the skin be kept active through frequent bathing, and if the diet be controlled in that way, and a large amount of exercise taken, the patient is not apt to suffer with it.

Dr. Howard called my attention to the salt treatment. I had a patient at that time with rheumatism, and I asked him about that, and he said he had always eaten large quantities of salt and did yet, but he suffered from rheumatism just the same. But that man did not take any active physical exercise; that man sat in the shop or office all the time; he ate heartily and ate a great deal of salt. You may take tobacco, and you may take all the rest of those things, and if you do not take into consideration your climate and the exercise of the human body, and the elimination through the ordinary channels, why, you won't control this diathesis at all. Dr. Olive's treatment is good: bicarbonate of sodium, bicarbonate of potassium or antikamnia, if it is simply the addition of the alkalies, ought to be about as good as any; but if a man will take proper exercise, and regulate his diet sufficiently, keep his skin open, he won't have the uric acid diathesis, or he will not suffer from those violent manifestations ot it.

But as sure as you live, there is a difference between rheumatism and ordinary gout, and uric acid does not explain it. You have to go somewhere else to get its explanation. This is a country that is free from gout, and there are not a great many rheumatics here, and the climate is largely responsible for it. And these old tobacco topers, that have soaked themselves in tobacco all their lives, would certainly have had the uric acid storms, or something of that kind, if there is not some other reason to explain it, as Dr. Blailock very happily says.

Dr. Hengst:—I have had quite a siege of uric acid diathesis for the last six or eight months in my own family, and of all the different treatments that have been recommended here, I think that I have tried everything that is laid down in the medical works, and the suggestions of all my medical friends, and I have never found anything yet that would eliminate it entirely. And I know that it could not have been caused by tobacco, unless it was absorbed, that produced it in this case. I tried alkalithia. I gave it a good, fair trial. It did benefit somewhat, but did not relieve entirely. And finally, on the suggestion of one of my brother physicians, I tried Buffalo Lithia Water. I tried that, and gave from one to two bottles of the Buffalo Lithia Water every day, and that appeared to relieve that head-thumping, the thumping of the back of the head down the back, and the nervous condition, more than anything that I have tried. Then I tried the tongalithia. That appeared to relieve a good deal. Finally I tried the lithia tablets of John Wyeth & Co., effervescent tablets, and let the patient take one of those five-grain tablets every two or three hours during the day, and she has had more relief from that than from any other medicines that I have tried, and now she is improving. Whenever she feels that acid coming on, she takes from four to five of those five-grain tablets, and in an hour or two, as soon as she gets the effect of it, that thumping and that nervous condition are entirely gone.

About producing uric acid, I think in this case it was entirely produced from nervous dyspepsia and from nervous exhaustion; that is, from nervous exhaustion was produced that uric acid diathesis. About a year ago was the first manifestation. It would come on occasionally, may be once or twice a month, and I would give from five to ten grains of salicylate of soda, and in the course of a day or two I would have her head entirely relieved; but that finally wore out; that would not have any effect at all. Then I gave her salicylate of quinia, and that would do it good, but that finally wore out, and the best results I have had were from the lithia tablets and tongalithia tablets combined. As Dr. Graves said, in the treatment we must regulate the diet. If she will eat two or three meals of butcher's meat, the back of her head will commence thumping in less than twenty-four hours, and then there is that peculiar taste, coated tongue, and whenever she gets up in the morning with coated tongue, she will know she is going to have some symptoms of her trouble during the day. And I thlnk that outdoor exercise, and an exclusive milk diet, and cold baths on retiring, with good friction, and things of that kind, will relieve the condition more than all other medicines put together; and if you will just keep the system from the accumulation of uric acid diathesis by the lithiates, the result will be—well, about the only thing that will relieve them at all will be any acid that will romove them.

The great trouble I have found was insomnia, just nervous,—couldn't sleep; and this continued thumping of the carotid. You could feel them thumping and see them thumping; and the peculiar heart trouble; she has made the remark again and again, "I feel like the heart turns over," and all those things come together. I think it is a marked case of uric acid diathesis, and I do not believe that smoking or the use of tobacco has much to do with it. About uric acid diathesis causing rheumatism and gout, I think it has something to do with it; but, as Dr. Graves says, there is very little gout around in this section of the country, and I know that there is considerable of uric acid diathesis.

Dr. Hunter:—I am sorry that I am almost sick to-night. I am nearer now an attack of pneumonia than I ever was in my life. Before commencing the discussion of the subject proper I want to thank Dr. Olive for his kindly mention of myself and my paper read before the State Medical Association of Texas, on the 23d day of April, 1895, at Dallas, in his most excellent paper. I want to say further, gentlemen, that I have not only had many compliments paid to me by physicians of Texas, more than a hundred; not only that, but I have received letters from all over these United States thanking me, hundreds of them, for bringing this important subject before the medical profession, not only of Texas, but of these United States, of uric acid as a cause of asthma. And I claim priority right here to-night of being the only man in the United States that ever read a paper upon that subject before any medical association. And while my motives were misjudged at the State Medical Association in bringing this uric acid theory of asthma before it, and recommending alkalitha as the best treatment, subsequent results, I am proud to say, have borne out that I was right in my convictions. Uric acid has figured as a factor in the causation of gout and rheumatism by most authors, both in the United States and Europe, for a number of years, but not until recent years has it figured as a cause of asthma in either country. The formation and causation of the uric acid diathesis has been discussed in the paper so thoroughly, and the literature of the subject presented so well, that I shall not have much to say upon that part of the subject. However, food appears to be the main factor in introducing uric acid into the system, especially a heavy meat diet, and the wines and malt liquors. Hence prophylaxis is very important in the treatment of the uric acid diathesis; a milk diet with vegetables would probably be the best prophylactic treatment after the blood has been cleared of the uric acid with medical agents. In my judgment lithia would be one of the best prophylactic agents when it comes to medicine. No, I do not consider lithia is a good solvent of the uric acid calculi after they have been formed, as these are mostly urate of calcium, but its office, on the other hand, is principally the prevention of the calculi by reason of the lithia combining with the uric acid to form a soluble urate of lithia, which is excreted, and hence does not become solidified in the pelvis of the kidneys or bladder, as happens when lithia is not present, and the calcium urate is formed. It is a fact observed clinically that these calculi disappear after a long time, or become rounded and less objectionable, under the action of lithia, after the urine becomes alkaline and is continued so, which is probably due to the fact that the calculi is very slightly dissolved, or the small pieces which may be worn off by attrition are disintegrated and dissolved by the alkaline character of the menstrum in which they are surrounded. The uric acid in crystallizing alone forms a very hard, sharp, insoluble crystal, and when lithia is present a soluble salt is formed; and hence no uric acid or urates remain in the system as a secretory product.

Now, when it comes to the curative treatment, nearly all the drugs of the materia medica have been recommended by some author, but to sum up, the iodides, the salicylates and the alkalies have been prescribed by more physicians and with better results, I think, than almost any other class of medicines. Piperozine has recently been praised by a good many as a uric acid solvent, while others say it is absolutely worthless in this trouble. (*Medical Record*, April 6, 1895, page 436.) In the same journal (September 14, 1895) Nojinkoff says if the kidneys are healthy piperozine will not cause albumenuria in small doses, but if the kidneys are diseased piperozine will increase albumenæria, as well as humaturea, even if given in small doses. Dr. Borig, in using this remedy upon patients undergoing treatment at the Weldangen Springs, has noted that it may produce albumenuria, as determined by the picric acid test. Therefore, I would consider it a dangerous medicine, as the kidneys are often more or less affected in the uric acid diathesis.

Now, Mr. President and gentlemen, while some authorities say lithia is a good uric acid solvent, and others say it is not, and some say that soda is, and others, that it is useless, we all know it to be a good alkaline preparation, especially the bicarbonate; but the bicarbonate of potassium is acknowledged by all to be a good uric acid solvent. In fact, some recent German authors say it is the only drug worth using in the uric acid diathesis. (*Medical Record*, April 6, 1895.) Now, in my judgment, a combination of the alkalies lithia, potassium and sodium, with the addition of that fine heart tonic and diuretic, caffeine, given in carbonic acid water, would be the best treatment. Most authors say that these salts act better when given in effervescence. Therefore alkalithia, which is an effervescent salt, and contains one grain of caffeine, five grains of carbonate lithia, and ten grains, each, of bicarbonate of soda and bicarbonate of potassium, a teaspoonful three or four times a day, would be the ideal treatment in my judgment. This combination does not form with uric acid an insoluble urate, but is the best eliminator of the uric acid, as well as the best uric acid solvent, in my judgment, known to the medical profession. I will just report a few cases of the uric acid diathesis treated by some of our best Texas physicians.

[Balance of Dr. Hunter's remarks omitted, as they were mainly in advocacy of a proprietary article in which he is interested. —Ed.]

Dr. Olive:—I desire to thank the members present for the kind remarks upon my paper; and as to the discussion that has followed, I certainly appeciate that very much, because I think the subject has been pretty thoroughly discussed, and discussed to the advantage of all of us.

Dr. Blailock starts out by saying that tobacco is not a cause of uric acid diathesis. It is a cause, as Dr. Howard explains, by producing the acid conditions of the blood, by rendering the uric acid insoluble and depositing it in the tissues. Dr. Blailock may as well have said that clouds do not produce rain because they pass over here every day and it does not rain. The conditions of the atmosphere are not such as to produce the necessary amount of condensation of the moisture in the atmosphere to produce the falling drops. The condition of the economy is not such as to produce the uric acid diathesis in every subject that uses tobacco. There may be things there to neutralize the effects of the tobacco. There may be alkalies in the system that render the uric acid soluble, retain it in a soluble condition in the blood and favor its elimination, its regular and constant elimination, and remove it from the system as it is formed. But it is a fact, that it converts ease into disease, it produces a weakened conditon of the system, a general feebleness of the system, and lessens the powers of resistance. It does that in addition to the deposition of the uric acid in the tissues. We might say that almost any cause that I have spoken of to-night will not produce it in every subject that partakes of those causes, Meat will not produce it in every man. Meat will only produce it in those whose condition is susceptible to the accumulation of uric acid, where other things are not equal to the emergency, and favor and continue to keep up the solubility of the uric acid and favor its elimination.

As to quinine, I do not know that I should answer that, because I did not mention it. I mention salicylate of quinia, and salicylate of cinchonidia; they are useful in this climate where there is any malaria in the system complicating the uric acid diathesis.

As to salt, Dr. Howard covers himself all over with glory when he claims originality in mentioning salt! Never heard of it before! I have heard of it and every other man has heard of it that has read Quain's dictionary of medicine, for the last fifteen years. And then harping on his *originality* in that. Of course it was not original with him. He got it from some good author, and then comes out here and claims originality.

Dr. Weathered asks a question that I think is pertinent. Uric acid in the blood causes contraction of the arterioles. Why not give coal tar preparations which dilate the arterioles? The coal tar preparations cause dilation of the arterioles. It eliminates the uric acid from the blood, throws it out, but it throws it out of the blood into the tissues; it does not eliminate it from the system. It does not favor the excretion of uric acid. That is the reason we do not give those things.

Dr. Graves of course, can cure his uric acid headaches with antikamnia, because it throws the uric acid out of the blood into the tissues, and it is only when the uric acid gets into the blood and produces this contraction of the arterioles that this uric acid headache follows. He only deposits the more of it in his tissues, and has perhaps a more severe headache the next time he has it. Now Haig's book, spoken of by Dr. Graves, I do not endorse in every respect; I do not endorse all he says, and I am not capable of endorsing a great deal that he says; but that book is well worth the careful, studious perusal of every medical practitioner. I have felt repaid an hundred times for the time I have devoted to Haig's work on "Uric Acid Diathesis." Dr. Graves spoke of his patient who ate large quantities of salt, and yet had the uric acid diathesis and had uric acid headaches, etc. Of course, if other conditions are favorable to the excessive accumulation of uric acid, a large quantity of salt might be necessary to make it soluble in the blood, and they might have uricacid headaches, or uric acid storms of various sorts, with the consumption of considerable quantities of salt. Dr. Graves speaks of bicarbonate of sodium and bicarbonate of potassium and antikamnia. Of course I explained my theory in regard to that before.

Dr. Hengst has never found anything to relieve the uric acid headache, I think, because he has not enjoined the proper amount of exercise, perhaps, and has not enforced the proper restrictions as to diet, and so forth, and at all times; has allowed the patient to go over the border line too often and indulge in articles of food or diet that could well be avoided, and perhaps would have been to the advantage of the patient. Perhaps he allowed liquors and wines to go to the house, and things of that sort.

DR. HENGST:—No, sir; Mrs. H. takes evercise every day, and I think in the last two or three months she has improved a great deal on account of being out of doors.

DR. OLIVE:—I agree with Dr. Hengst that it may be due to dyspepsia and nervous exhaustion. And I fully agree with him in his opinion that the medical treatment is not of the first importance, but that the diet and exercise and so on, and the consumption of large quantities of hot water, and not cold baths as much as hot favor the good free action of the skin. Use the hot bath and eliminate as much of the poisonous substances through the skin as possible, and you will have happier results, I think, than with cold baths.

DR. HENGST:—I gave the hot baths right along, but when I changed off and gave her cold sea baths, there were better results.

DR. OLIVE:—Well, some people will bear the cold better than the hot.

On motion a vote of thanks was tendered Dr. Olive for his paper.

